# Usefulness of brain FDG PET/CT imaging in pediatric patients with suspected autoimmune encephalitis from a prospective study

**DOI:** 10.1007/s00259-021-05649-w

**Published:** 2021-12-23

**Authors:** Yafu Yin, Jing Wu, Shuqi Wu, Suyun Chen, Weiwei Cheng, Ling Li, Hui Wang

**Affiliations:** 1 Department of Nuclear Medicine, Yangpu District, Xinhua Hospital Affiliated To Shanghai Jiao Tong University School of Medicine, Kongjiang Road 1665, Shanghai City, 200092 China; 2 Department of Pediatric Neurology, Yangpu District, Xinhua Hospital Affiliated To Shanghai Jiao Tong University School of Medicine, Kongjiang Road 1665, Shanghai City, 200092 China

**Keywords:** Autoimmune encephalitis, FDG PET/CT, Lobar hypometabolism, Suspected AE

## Abstract

**Purpose:**

Early diagnosis and treatment are of paramount importance for pediatric patients with autoimmune encephalitis (AE). The aim is to evaluate the usefulness of FDG PET/CT in pediatric patients with suspected AE from a prospective study.

**Methods:**

The prospective study was conducted over a period of 23.5 months from May 14, 2019, to April 30, 2021. All patients (< 18-year-old) were hospitalized at the department of pediatric neurology and met the criteria of clinical suspected AE. The children underwent the tests of blood samplings, CSF, EEG, MRI, and ^18^F-FDG PET/CT. The criteria for FDG PET/CT diagnosis of AE were large lobar hypometabolism with or without focal hypermetabolism found on PET/CT. The clinical final diagnosis of AE includes seropositive and seronegative AE based on the diagnostic criteria.

**Results:**

One hundred four pediatric inpatients (57 boys, 47 girls) were included, of which 58 children were diagnosed with AE (seropositive, 16; seronegative, 42), 45 children were diagnosed with non-AE, and one boy remained indeterminate diagnosis. Large lobar hypometabolism was found in 61 children, of which 54 (88.5%) children were finally diagnosed with AE. The sensitivity, specificity, and accuracy of FDG PET/CT for diagnosis of AE were 93.1%, 84.4%, and 89.3%, respectively, with a positive predictive value of 88.5% and a negative predictive value of 90.5%. The most common involved with hypometabolism was the parietal lobe, followed by occipital and frontal lobes, finally the temporal lobe on PET/CT in children with AE.

**Conclusion:**

Brain FDG PET/CT imaging has high specificity, sensitivity, and accuracy for diagnosis of AE in clinical suspected AE children.

Trial registration.

Clinical Trials.gov. NCT02969213. Registered 17 October 2016.

## Introduction

Autoimmune encephalitis (AE) generally refers to a group of severe inflammatory brain disease due to abnormal autoimmunity mechanism. The archetypical syndrome of AE includes variable combinations of neuropsychiatric and behavioral changes, psychosis, seizures, memory and cognitive deficits, abnormal movements, dysautonomia, and a decreased level of consciousness [1]. AE is one of the most challenging clinical conditions to diagnose because of the heterogeneity in disease manifestations and diagnostic test results, overlap in clinical presentations between AE, other inflammatory brain diseases, metabolic diseases, and psychiatric disorders. It is especially difficult for children because of the complexity of normal behavioral changes during childhood and the limited capacity of younger children to describe their symptoms. Early diagnosis is paramount for pediatric patients with AE because early initiation of immunotherapy is associated with a better prognosis [2–8], even severely affected children can have profound improvement when getting early and aggressive treatment [9, 10], otherwise worsened prognosis and even permanent neurocognitive deficits happened [11].

In the initial stage, the diagnostic criteria for AE were dependent on autoantibody testing and response to immunotherapy, while, depending on autoantibody testing, the diagnosis may significantly delay treatment initiation. Moreover, autoantibody testing is not readily available at most institutions, and there might be some autoantibodies have not yet been identified by now. In 2016, an international group developed diagnostic criteria for early diagnosis of AE in adults, which included the diagnoses of possible, probable, and definite AE to reduce the delay in initiation of therapy due to waiting for autoantibody testing results [12]. The items of criteria described in the report were composed of clinical symptoms, cerebrospinal fluid (CSF) sampling, serum sampling, electroencephalogram (EEG), and MRI. It is a real pity that ^18^F-FDG PET/CT was not recommended in the criteria as a diagnostic tool for possible AE or seronegative probable AE. In fact, a significant percentage of AE, mostly more than half, brain MRI shows normal or only non-specific changes [13]. ^18^F-FDG PET/CT shows a much greater sensitivity for detecting an underlying abnormality than MRI in patients with AE [14–16]. Abnormal brain metabolism detected on FDG-PET/CT is most often in early stage of AE, compared to initial EEG, MRI, and CSF studies in inpatients with AE [17].

So far, many works have described the characteristic findings supporting the diagnostic value of ^18^F-FDG PET/CT in AE [18–24]. PET imaging was not included in the diagnostic criteria mostly because there was short of robust evidence to accelerate the comprehension and validation of ^18^F-FDG PET in the emerging field of AE [25], and especially due to the lack of prospective studies. The data is even more limited in pediatric AE, as noted in recently published clinical approach to the diagnosis of AE in pediatric patient [26].

In this study, a prospective study was conducted and aimed to evaluate the usefulness of FDG PET/CT in pediatric patients with clinical suspected AE.

## Methods

### Study design and participants

This prospective study was conducted over a period of 23.5 months from May 14, 2019, to April 30, 2021, in Xinhua Hospital affiliated to Shanghai Jiao Tong University School of Medicine. The pediatric inpatients (< 18 year-old), with the following criteria, were considered clinical suspected AE and included: (1) clinical symptoms: seizures, neuropsychiatric changes (e.g. memory and cognitive deficits, behavioral changes, psychosis), movement disorders and dysautonomia, and (2) at least one of the following abnormalities: CSF pleocytosis; CSF-specific oligoclonal bands or elevated CSF IgG index; elevated erythrocyte sedimentation rate, complement protein, antinuclear antibody, immunoglobulin, lymphocyte count, and inflammatory factors in serum sampling; MRI features suggestive of encephalitis; abnormal EEG; positive etiologic test; paraneoplastic antibody.

All the enrolled patients underwent brain ^18^F-FDG PET/CT scanning and neuronal autoantibody test in the serum and CSF samplings. Based on the further findings, the pediatric neurologist gave the children further immunotherapy as appropriate [2]. Exclusion criteria were as follows: (1) refuse to perform PET/CT scanning or fail to finish PET/CT scanning, (2) refuse further examination or immunotherapy under no definite diagnosis, and (3) infant with age less than one-year-old.

The criteria of clinical final diagnosis of AE, for the enrolled suspected AE patients, include the following:

### Seropositive AE

The autoimmune neuronal antibodies were identified in the serum and/or CSF samplings.

### Seronegative AE

The diagnosis was made when all three of the following criteria have been met: (1) none of autoimmune neuronal antibody was identified in the serum or CSF sampling, (2) ≥ 1 feature present: CSF-specific oligoclonal bands; intrathecal synthesis of immunoglobulin or elevated CSF IgG index; CSF pleocytosis (> 5 cells) or elevated protein; brain MRI abnormalities suggestive of AE; effective immunotherapy, and (3) reasonable exclusion of alternative causes.

### Brain ^18^F-FDG PET/CT imaging

Brain ^18^F-FDG PET/CT imaging was performed according to the routine institutional procedures. In brief, all patients fasted from food and sugary drinks for at least 4 h prior to intravenous injection of approximately 25–185 MBq ^18^F-FDG based on 3.7 MBq/kg body weight. All patients rested in a quiet and dim room before and after injection. About 50 min following injection, a brain PET/CT scanning was performed for 10 min on a Biograph mCT-64 scanner (Siemens, Erlangen, Germany).

### Imaging analysis

All PET/CT images were independently assessed and reviewed by two nuclear medicine physicians with extensive experience with brain FDG PET/CT imaging.

The criteria for FDG PET/CT diagnosis of AE and non-AE: (1) large lobar hypometabolism with or without focal hypermetabolism found on PET/CT was defined as AE; (2) focal hypometabolism which is consistent with a clinical epileptic seizure, even if combined with extensive area of hypometabolism around, was defined as an epileptogenic focus, or hypometabolism could be explained by tumor or other disease or not were all classified as non-AE; (3) no obvious abnormalities found on PET/CT were classified as non-AE. Large lobar hypometabolism means extensive decreased glucose metabolism in bilateral lobes or more than one unilateral lobe.

### Statistical analysis

Data analysis was performed using the IBM SPSS Statistics 25 software (USA: IBM Corp). Descriptive statistics included the frequency (percentage) for categorical variables and median for continuous variables. Chi-square, fisher’s exact, and two-tailed *t* student test were used to compare the clinical, laboratory, and imaging features between the groups of pediatric patients as appropriate. All tests were two-tailed and *p* < 0.05 was considered statistically significant.

## Results

### Patient demographics

In total, 104 pediatric patients (57 boys, 47 girls, median age 7 years, range 1–17 years) were recruited in this prospective study. The clinical characteristics of the included children are summarized in Table 1, except for one boy whose diagnosis was indeterminate.

**Table 1:** The clinical characteristics of 103 children with definite diagnosis

Characteristic	Total	AE		Non-AE	p**	p***
		**Seropositive**	**Seronegative**			
Number	103	58		45	–	–
		16 (27.6%)	42 (72.4%)			
Median age (range), years	7 (1–17)	7 (1–17)		7 (1–15)	0.409	0.927
		6.5 (2–17)	7 (1–13)			
Gender						
Female	47 (45.6%)	30 (51.7%)		17 (37.8%)	0.311	0.169
		10 (62.5%)	20 (47.6%)			
Male	56 (54.4%)	28 (48.3%)		28 (62.2%)		
		6 (37.5%)	22 (52.4%)			
Clinical syndromes						
Seizures	61 (59.2%)	32 (55.2%)		29 (64.4%)	0.280	0.342
		7 (43.8%)	25 (59.5%)			
Neuropsychiatric changes	62 (60.2%)	41 (70.7%)		21 (46.7%)	0.017	0.013
		15 (93.8%)	26 (61.9%)			
Movement disorders	4 (3.9%)	3 (5.2%)		1 (2.2%)	1.000	0.630
		1 (6.3%)	2 (4.8%)			
Dysautonomia	13 (12.6%)	11 (19.0%)		2 (4.4%)	1.000	0.036
		3 (18.8%)	8 (19.0%)			
Infection	95 (92.2%)	55 (94.8%)		40 (88.9%)	0.819	0.264
		15 (93.8%)	40 (95.2%)			
CSF*	39 (40.6%)	34 (58.6%)		5 (12.5%) (7 untested)	0.156	0.000
		7 (43.8%)	27 (64.3%)			
Erythrocyte sedimentation rate	18 (18.4%)	12 (21.1%)		6 (14.6%) (4 untested)	0.238	0.418
		5 (31.3%)	7 (17.1%) (1 untested)			
Autoantibody^#^	24 (24.2%)	20 (34.5%)		4 (9.8%) (4 untested)	0.359	0.005
		7 (43.8%)	13 (31.0%)			
Cellular immunity	95 (97.9%)	56 (98.2%)		39 (97.5%) (5 untested)	0.529	0.799
		16 (100%)	40 (97.6%) (1 untested)			
Inflammatory factors	77 (81.1%)	47 (85.5%)		30 (75.0%) (5 untested)	0.05	0.199
		16 (100%)	31 (79.5%) (3 untested)			
EEG	67 (65.0%)	38 (65.5%)		29 (64.4%)	0.359	0.910
		9 (56.3%)	29 (69.0%)			
MRI	23 (22.3%)	11 (19.0%)		12 (26.7%)	0.438	0.352
		2 (12.5%)	9 (21.4%)			

^*^CSF abnormalities includes CSF-specific oligoclonal bands; intrathecal synthesis of immunoglobulin or elevated CSF IgG index; CSF pleocytosis (> 5 cells) or elevated protein

^**^This *p* value is the chi-square test for seropositive and seronegative AE groups

^***^This *p* value is the chi-square test for AE and non-AE groups

^**#**^The term autoantibody here refers to the antibodies that are associated with connective tissue disorders

### Final definite diagnosis

Finally, 58 children were diagnosed with AE, of which 16 children were seropositive and 42 were seronegative AE. Forty-five children were diagnosed with non-AE, and one child remained indeterminate diagnosis, who was an 11-year-old boy with neuropsychiatric changes, infection, negative results of CSF, EEG, and MRI, and no response to immunotherapy.

Sixteen seropositive AE children presented with positive neuronal autoantibody test for anti-NMDAR (7 children), anti-Ri (2 children), anti-MOG (2 children), anti-Hu and anti-Amphiphysin (1 girl), anti-DPPX (1 girl), anti-GQ1b (1 boy), anti-AMPAR1 (1 boy), and anti-Recoverin (1 girl).

Among 45 non-AE patients, 23 children (51%) were diagnosed with epilepsy, 9 children were diagnosed with mental retardation, 4 were tic disorder, 3 were headache, and 6 were diagnosed with brain tumor, Niemann-Pick disease, postencephalitis, somatization disorder, syringomyelus, and hydrocephalus, respectively.

### Clinical manifestation

The most common manifestations of the recruited children with definite diagnosis were seizures (61 children, 59.2%) and neuropsychiatric changes (62 children, 60.2%). Neuropsychiatric changes were more often presented in AE children (70.7%) than non-AE (46.7%) (*p* < 0.05). Twenty-six children presented with both seizures and neuropsychiatric changes, of which 6 children were seropositive AE, 13 were seronegative AE, and 7 were non-AE. Seizures happened with neuropsychiatric changes more often in AE children (19/32) than non-AE (7/29) (*p* < 0.05). Less common manifestations were dysautonomia (13 children, 12.6%) and movement disorders (4 children, 3.9%). Dysautonomia was relatively more often presented in AE children in this study (*p* < 0.05) (Table 1).

### Clinical examination

Infection, cellular immunity, and inflammatory factors were very common in AE children with a high positive rate of 94.8%, 98.2%, and 85.5% (Table 1), respectively, which were slightly higher than that in non-AE children (88.9%, 97.5%, and 75.0%), but no significant difference reached.

The CSF abnormalities, including CSF-specific oligoclonal bands positive, intrathecal synthesis of immunoglobulin or elevated CSF IgG index, CSF pleocytosis (> 5 cells) or elevated protein, were more often positive in AE than non-AE children (58.6% & 12.5%, *p* < 0.05).

The autoantibodies associated with connective tissue disorders and erythrocyte sedimentation rate were less often positive in AE and non-AE children (34.5%, 21.1% & 9.8%, 14.6%); the difference of the former between groups reached statistical significance (*p* < 0.05).

In non-AE children, MR imaging showed non-specific abnormalities in 8 cases, brainstem encephalitis, meningocephalitis, tuberous sclerosis, and hydrocephalus in one case each. In AE children, MR showed non-specific abnormalities in 7 cases, pituitory Rathke cyst in 2 cases, encephalitis and focal cortical dysplasia in one case each.

EEG abnormalities were common in both AE and non-AE children (65.5% & 64.4%); no significant difference reached (*p* < 0.05).

### Application of newly published diagnostic criteria for pediatric AE

The newly published diagnostic criteria for pediatric AE were developed by the Autoimmune Encephalitis International Working Group in 2020 [26]. The proposed classification criteria for possible pediatric AE, probable antibody-negative pediatric AE, and definite antibody-positive pediatric AE were provided in Table 2 [26]. Among seropositive AE patients, 10 (62.5%) children fulfilled criteria for definite antibody-positive AE; the other 6 children did not meet the criteria mostly due to onset of neurologic and/or psychiatric symptoms over > 3 months. Among seronegative AE patients, 13 (31.0%) children fulfilled criteria for possible AE, 4 (9.5%) fulfilled criteria for probable antibody-negative AE, and 25 (59.5%) children did not meet the criteria. The proportion of pediatric patients meeting the latest diagnostic criteria was very low, mainly because of the long onset of symptoms, normal or non-specific CSF and MRI findings, and the absence of a brain biopsy.

**Table 2:** The proposed classification criteria by AE International Working Group [26]

Categorical features of AE	Possible AE	Probable antibody-negative AE	Definite antibody-positive AE
**Acute or subacute onset:** Onset of neurologic and/or psychiatric symptoms over ≤ 3 mo in a previously healthy child	Yes	Yes	Yes
**Clinical evidence of neurologic dysfunction:** Altered mental status/level of consciousness or EEG with slowing or epileptiform activity (focal or generalized) Focal neurologic deficits Cognitive difficulties Acute developmental regression Movement disorder (except tics) Psychiatric symptoms Seizures not explained by a previously known seizure disorder or other condition	≥ 2 features present	≥ 2 features present	≥ 2 features present
**Paraclinical evidence of neuroinflammation:** CSF inflammatory changes (leukocytosis > 5 cells/mm^3^ and/or oligoclonal banding) MRI features of encephalitis Brain biopsy showing inflammatory infiltrates and excluding other disorders	Not available	≥ 1 features present	≥ 1 features present
**AE serology:** Presence in serum and/or CSF of well-characterized autoantibodies associated with AE	Not available	No	Yes
**Exclusion of other etiologies:** Reasonable exclusion of alternative causes, including other causes of CNS inflammation	Yes	Yes	Yes

#### Brain FDG PET/CT imaging

All the patients underwent brain FDG PET/CT scanning, of which 9 children underwent scanning twice. The results of 103 primary brain PET/CT imaging were listed in Table 3, except for the boy with indeterminate diagnosis.

**Table 3:** The results of brain PET/CT imaging

PET/CT		AE (58)		Non-AE (45)	p**	p***
		**Seropositive(16)**	**Seronegative(42)**			
AE (61)	Lobar A^*^ (23)	21 (36.2%)		2 (4.4%)	0.031	0.000
		2 (12.5%)	19 (45.2%)			
	Lobar B^*^ (38)	33 (56.9%)		5 (11.1%)	0.036	0.000
		13 (81.3%)	20 (47.6%)			
Non-AE (42)	Focal foci (19)	2 (3.4%)		17 (37.8%)	0.479	0.000
		1 (6.3%)	1 (2.4%)			
	Normal (23)	2 (3.4%)		21 (46.7%)	1.000	0.000
		0	2 (4.8%)			

^*^Lobar A stands for lobar hypometabolism without focal hypermetabolism; Lobar B stands for lobar hypometabolism with focal hypermetabolism

^**^This *p* value is the chi-square test for seropositive and seronegative AE groups

^***^This *p* value is the chi-square test for AE and non-AE groups

**Fig. 1:**
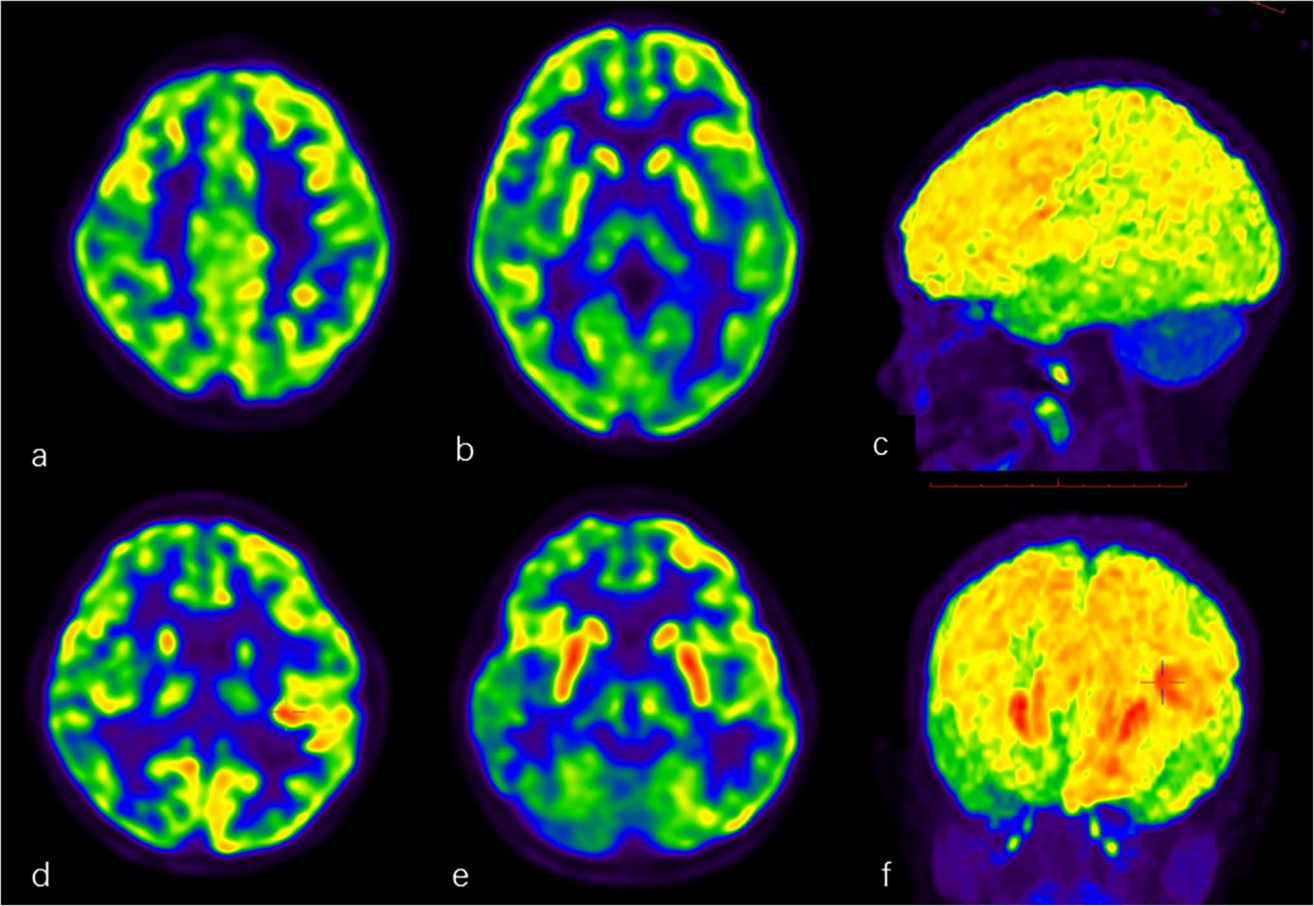
Top row: A 4-year-old girl presenting with fewer words and personality changes. Anti-MOG antibody test was positive. Brain ^18^F-FDG PET/CT showed large-scale lobar hypometabolism in bilateral parietal and occipital lobes without focal hypermetabolism (**a**, **b**, **c**). Bottom row: An 8-year-old girl presented with seizures, personality, and cognitive changes for more than 3 months, who was finally diagnosed with anti-NMDAR AE. Brain ^18^F-FDG PET/CT showed large-scale hypometabolism in bilateral parietal, occipital, temporal, and right frontal lobes (**d**, **e**, **f**) with focal hypermetabolism in left frontal and fronto-temporal lobe (the cross: **d**, **f**) and bilateral basal ganglia (**e**, **f**). **c**, **f** MIP map of FDG PET

**Fig. 2:**
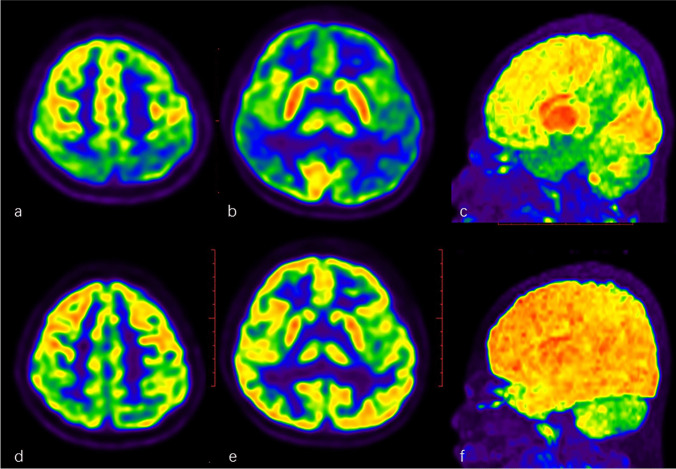
A 7-year-old boy with a 6-year history of seizures and developmental retardation was finally diagnosed with seronegative AE. Brain ^18^F-FDG PET/CT showed large-scale hypometabolism in bilateral parietal, temporal, and frontal lobes with focal hypermetabolism in basal ganglia (**a**, **b**, **c**). 2.5 months later, the second brain ^18^F-FDG PET/CT scanning indicated the significant recovery of the abnormal metabolism (**d**, **e**, **f**) after immunotherapy, consistent with the improvement of clinical symptoms. **c**, **f** MIP map of FDG PET

**Fig. 3:**
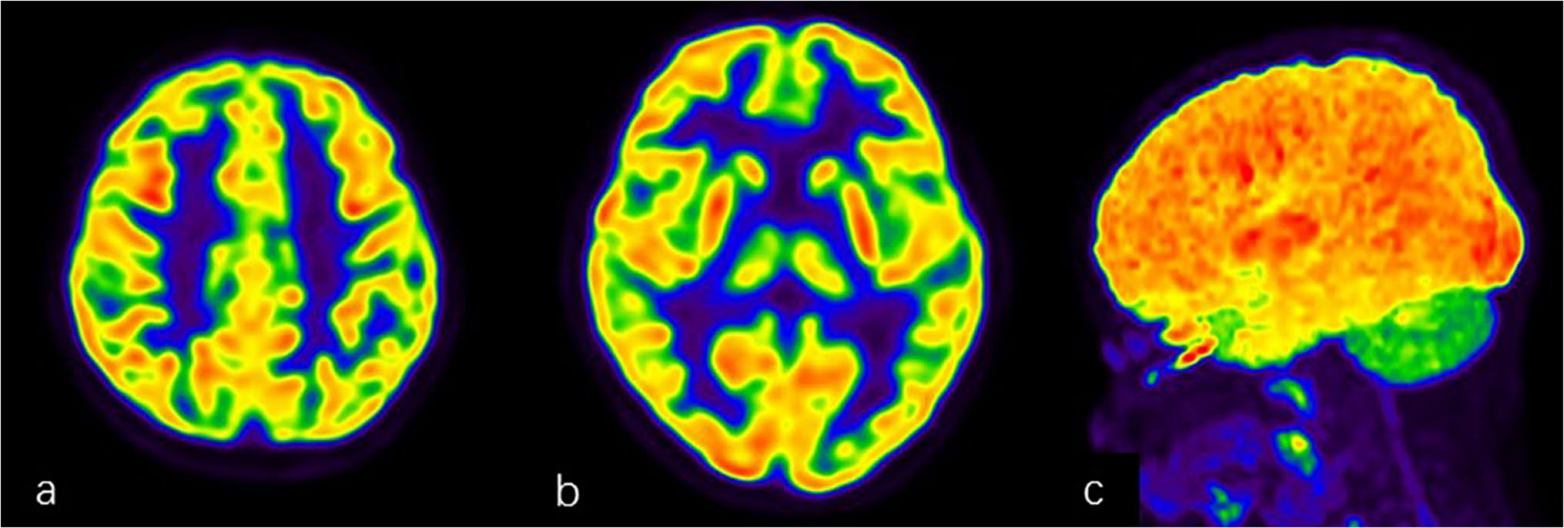
A 7-year-old boy with a 5-year history of seizures presented with normal brain ^18^F-FDG PET/CT imaging. **a**, **b** Cross-sectional image of FDG PET. c:MIP map of FDG PET

**Fig. 4:**
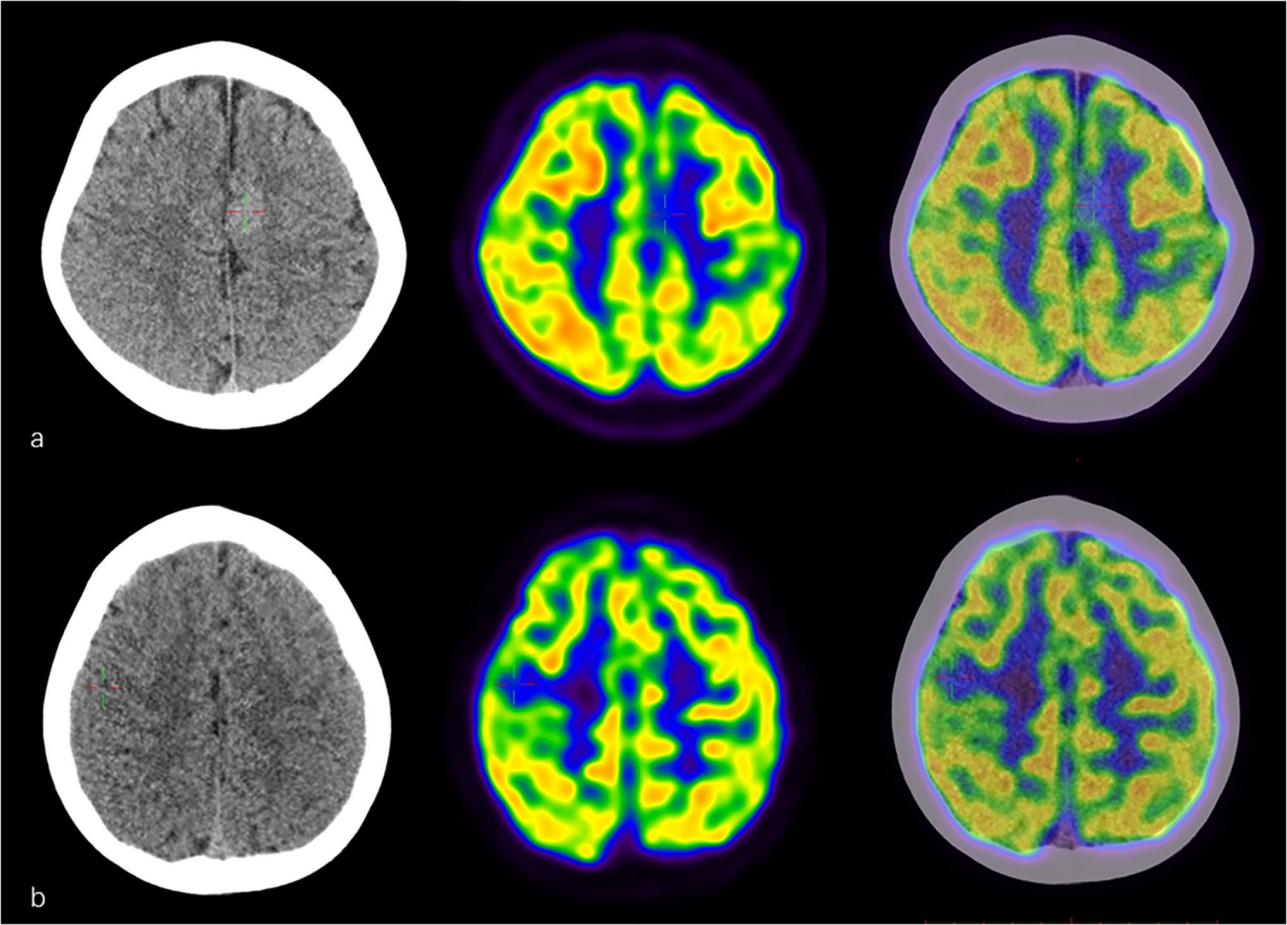
**a** A 6-year-old boy with a 9-month history of seizures. Brain ^18^F-FDG PET/CT showed focal hypometabolism in left frontal lobe (the cross) with high density on CT correspondingly (the cross). The boy was finally diagnosed with frontal epilepsy. **b** A 6-year-old girl with a 4-month history of seizures. Brain ^18^F-FDG PET/CT indicated focal hypometabolism in right central sulcus mainly in the parietal lobe (the cross) with no abnormality found on CT correspondingly (the cross). The girl got well recovery after immunotherapy and was finally diagnosed with seronegative AE

Among 103 children with definite diagnosis, large lobar hypometabolism with or without focal hypermetabolism (Figs. 1 and 2) was found on PET/CT in 61 children, of which 54 (88.5%) children were eventually diagnosed with AE. Normal brain imaging (Fig. 3) was found in 23 children, of which 2 (8.7%) children were diagnosed with AE. Focal foci with hypometabolism (Fig. 4) were found in 19 children, of which 2 (10.5%) children were diagnosed with AE finally. Among the children with focal foci, epileptogenic foci were found on PET/CT in 10 children. The sensitivity, specificity, and accuracy of FDG PET/CT for diagnosis of AE were 93.1% (54/58), 84.4% (38/45), and 89.3% (92/103), respectively, with a positive predictive value of 88.5% (54/61) and a negative predictive value of 90.5% (38/42). The boy with indeterminate diagnosis showed lobar hypometabolism with focal hypermetabolism.

In total, lobar hypometabolism with focal hypermetabolism was found on PET/CT in 38 children with definite diagnosis, of which 33 (86.8%) children were diagnosed with AE composed of 8 focal cortical hypermetabolism and 27 hypermetabolism in basal ganglia. Among children involved with lobar hypometabolism without focal hypermetabolism, 91.3% (21/23) were diagnosed with AE. In the 58 AE children, the most common involved with hypometabolism was the parietal lobe (55, 94.8%), followed by the occipital (35, 60.3%) and frontal lobes (34, 58.6%), finally the temporal lobe (22, 37.9%) on PET/CT.

In seronegative AE children, abnormal metabolism involved 3 children in one lobe (bilateral) (7.1%), 16 children in two lobes (38.1%), 12 children in three lobes (28.6%), 8 children in four lobes (19.0%), 15 children in basal ganglia (35.7%). In seropositive AE children, abnormal metabolism involved 1 child in one lobe (bilateral) (6.3%), 5 children in two lobes (31.3%), 4 children in three lobes (25.0%), 5 children in four lobes (31.3%), and 12 children in basal ganglia (75.0%).

Four children with AE showed normal or focal hypometabolism on PET/CT, three of which mainly presented with developmental retardation, especially in language, and had a longer course than one year. One child presented with seizure; PET/CT showed focal hypometabolism in right central sulcus mainly in the parietal lobe (Fig. 4).

Six children had the repeated brain PET/CT scanning 1 to 7 months after immunotherapy, hypometabolism got well recovered (Fig. 2). Three patients had the scanning shortly (19 days to 2.5 months) after therapy, hypermetabolism of basal ganglia appeared or got more obvious and more extensive hypometabolism presented. All these 9 patients were diagnosed with seronegative AE.

### Brain FDG PET/CT imaging improved the diagnosis of AE by newly published diagnostic criteria for pediatric AE

Four children who met the criteria for probable antibody-negative AE and 10 children who met the criteria for definite antibody-positive AE were all diagnosed with AE by brain FDG PET/CT imaging. Twelve of 13 children who fulfilled the criteria for possible AE were diagnosed with AE by FDG PET/CT imaging, and 28 of 31 children who did not meet the proposed criteria were diagnosed with AE by FDG PET/CT (Table 4).

**Table 4:** FDG PET/CT improved diagnosis of AE based on proposed classification criteria by AE International Working Group

Final diagnosis	Classification diagnosis by AE International Working Group			
	**Possible AE**	**Probable antibody-negative AE**	**Definite antibody-positive AE**	**Not met**
**Seropositive AE (16)**	-	-	10	6
**Seronegative AE (42)**	13	4	-	25
FDG PET/CT* (54)	12(92.3%)	4(100%)	10(100%)	28(90.3%)

^*^Abnormal metabolism on FDG PET/CT was diagnosed with AE

## Discussion

For AE, as a group of treatable acquired central nervous system disorders mostly affecting children and young people, the immunosuppression therapy, especially the early initiation of treatment, is crucial for optimal clinical outcome. In recent decade, much advances have emerged in this field, but a great part of children with suspected AE was still misdiagnosed in the initiation and missed the best time starting treatment, mostly due to having unidentified autoantibodies [2], who might be seronegative or delayed positive autoantibody test, and not meet the existing criteria, which depends on the abnormal findings of serum or CSF sampling, EEG and MRI [12, 26]. Most of the suspected AE children were with various unspecific symptoms, and as has been widely reported, a large proportion of suspicions have normal or non-specific findings of serum or CSF sampling [27, 28], EEG [17], and MRI [13, 14].

Since the publication of a clinical approach to diagnosis of AE in the journal of Lancet Neurology in 2016, a large number of studies on FDG PET, almost all were retrospective, have been reported and indicated that FDG PET is an important biomarker for suspected AE [18]. These studies showed a much greater sensitivity for detecting abnormalities in AE than MRI [14] and other tests currently included in consensus criteria for the diagnosis of AE [17]. The predominant abnormality on brain FDG PET imaging was lobar hypometabolism, which mostly involved parietal and occipital lobes, some with hypermetabolism, mainly in basal ganglia and partial cortical lobe [14, 17, 22, 29–31].

Based on the valuable metabolic abnormalities on brain FDG PET/CT imaging from the retrospective studies in AE patients, we designed this prospective study for suspected AE children, and the diagnostic criteria for FDG PET/CT were extensive hypometabolism in more than one lobe or one lobe with bilateral involved with or without focal hypermetabolism. The results indicated that ^18^F-FDG PET/CT had high specificity, sensitivity, and accuracy in diagnosing AE for suspected pediatric AE. FDG PET/CT showed large lobar hypometabolism involved mostly in the parietal lobe, occipital and frontal lobes followed, and temporal lobe last in AE children. More than 85% of children with AE showed abnormal metabolism involved in at least two lobes. Nearly half of the children with AE showed hypermetabolic basal ganglia, and 13.8% showed hypermetabolism in focal cortex on FDG PET/CT, which was similar to the previous report [22, 29, 30]. In total, nearly up to 60% of children with AE showed lobar hypometabolism with focal hypermetabolism.

In this prospective study, the positive rate of FDG PET/CT in AE children was 93.1% on visual assessment, which was similar to the reported data (85–100%) [14, 17, 30]. In fact, our positive rate was only about lobar abnormalities excluding focal hypometabolism; in this point, it was different from those reported retrospective studies. Previous studies have shown that the autoimmune antibodies can directly disrupt the synaptic function of affected neurons [32, 33], which can explain the lobar hypometabolism in AE patients. Anyway, hypometabolism can occur in many diseases, such as stroke, tumor, and epilepsy. Stroke and tumor usually present with regional decreased glucose metabolism and unilateral foci and could be easily identified by CT or MR. The patients with epilepsy mostly present with unilateral focal hypometabolism in interictal stage and focal hypermetabolism in ictal stage, although some cases were accompanied with extensive area of hypometabolism in addition to bilateral thalamic and cerebellar hypometabolic changes [34, 35]. Large lobar hypometabolism, more than one lobe, at least bilateral lobes, seemed more commonly observed in AE patients, which could be considered the specific findings on FDG PET/CT for patients with suspected AE. Of course, hypermetabolism in basal ganglia and/or cortex is also a specific finding accompanied with lobar hypometabolism, which happened in more than half cases.

CSF sampling, EEG, and MRI were also analyzed in this study. Although there was significant difference between AE and non-AE, the positive rate of CSF abnormalities was only 58.6% in AE children, much lower than FDG PET/CT with 93.1%. As reported, 56% of patients with AE could have CSF pleocytosis, but about 30% of patients with AE had a “normal” CSF, which is considered the key element of proposed diagnostic criteria for possible AE [27, 28]. The positive rate of EEG was over 60% in both AE and non-AE groups.

In our study, the positive rate of MRI was lower than some reports, and no specific AE abnormalities were found except for one girl who showed specific abnormal changes on MRI, but the girl was diagnosed with brain tumor in the end. Usually, if the children presented specific abnormalities of AE on MRI and got the right diagnosis and treatment, few would visit our hospital. This could, at least in part, explain the low finding rate of specific abnormalities on MRI in this study. Nevertheless, all the reported data indicated that the positive rate of specific findings on MRI was very low in AE patients [14–16], that is to say that most of the hypometabolism involved regions did not show abnormalities on MRI, suggesting the dysfunction of neurons was in the absence of structural disorders. As reported in this study, extensive hypometabolism in multiple lobes likely reflects widespread impairment of neuronal activity [36], exactly synaptic activity in AE, based on glucose consumption at synapses increasing in proportion of neuronal activity [34] and the evidence of autoimmune antibodies directly disrupting the synaptic function of affected neurons [32, 33].

Seizures were found in 59.2% recruited children with suspected AE, and 55.2% of AE children presented with seizures in this study. As reported, seizures were thought to be the predominant manifestation in AE [26]. In a very recent meta-analysis, the overall incidence of AE patients with seizures was 42%, and in the anti-NMDAR encephalitis patients, the rate increased to 73% [37]. Anti-NMDAR encephalitis is the most common type of AE in pediatric patients. Among the 104 recruited children, 23 children, nearly half of non-AE patients, were eventually diagnosed with epilepsy. FDG PET/CT found the epileptogenic foci in 10 children successfully. In this study, neuropsychiatric changes were found at a higher rate of 70.7% than seizures in AE children. The children presenting with both seizures and neuropsychiatric changes were more likely diagnosed with AE compared with non-AE.

In this study, 9 children underwent the repeated FDG PET/CT scanning after immunotherapy, two-thirds of them demonstrated good recovery on PET imaging 1 to 7 months after therapy, showing excellent evidence of the recovery of neuronal synaptic function. One-third showed more obvious abnormal metabolism on FDG PET imaging after a short time, which might be due to the different clinical course. Some studies showed the variable brain metabolic patterns might be associated with the different clinical course; the cortical hypometabolism might be more extensive in early recovery phase [29].

In our study, 31 pediatric patients with AE did not meet the latest diagnostic criteria of AE in the pediatric patient [26], while 90.3% of them were correctly diagnosed by brain FDG PET/CT imaging. Similarly, 92.3% of patients who fulfilled the criteria for possible pediatric AE were correctly diagnosed with AE by brain FDG PET/CT imaging. The most common reasons that more than half of pediatric patients with AE did not meet the proposed diagnostic criteria were long onset of symptoms, normal or non-specific MRI and CSF findings, and the absence of brain biopsy. Similar reports about the application of proposed diagnostic criteria were published in adult AE. Giordano et al. [38] reported that 30.3% of included adult AE patients did not meet the criteria for definite or probable AE. Another report showed nearly 15% patients did not meet the criteria for possible AE, and of the patients with anti-NMDAR encephalitis, 17% did not fulfil the diagnostic criteria for possible AE, and 25% failed to meet the criteria for probable anti-NMDAR encephalitis [39]. Based on our study, it is suggested that when pediatric patients with suspected AE failed to meet the criteria for probable or definite AE, especially with normal or non-specific CSF and MRI findings and the absence of a brain biopsy, brain FDG PET/CT imaging is strongly recommended.

Some limitations in this study need to be mentioned. First, the recruited children were all at least one-year-old. There was no data analyzed about infants younger than one-year-old due to infant brain being in development with variable glucose metabolism in the cortex. Second, there was no quantitative statistical software adopted due to no normal children’s brain FDG PET imaging template. Although some retrospective studies reported using the adult brain FDG PET template, it seemed not precise and critical meaningful. The normal template of children is really hard or almost impossible to obtain for ethical consideration.

## Conclusion

This prospective study indicated that brain FDG PET/CT was very useful for clinically suspected AE children, with high specificity, sensitivity, and accuracy for diagnosis of AE. The typical findings, large lobar hypometabolism, mostly accompanied with hypermetabolism in basal ganglia and/or focal cortex, on FDG PET/CT were strong supporting criteria for AE diagnosis in suspected children. Overall, brain FDG PET/CT should be considered to be included in the diagnostic criteria for AE in children, especially for those with negative neuronal autoantibodies, and normal CSF and non-specific brain MRI findings, who failed to meet the criteria for probable AE.

## Data Availability

The datasets generated during and/or analyzed during the current study are available from the corresponding author on reasonable request.
